# Showing the Non-Existence of Two Changes of Color of the Blood

**Published:** 1868-06

**Authors:** R. King Browne


DENTAL REGISTER.

Vol. XXII.] JUNE, 1868. [No. 6.

### Original Communications.

### SHOWING THE NON-EXISTENCE OF TWO CHANGES OF COLOR OF THE BLOOD.

BY PROF. R. KING BROWNE.

It has never been doubted that the dark color of the blood is due to carbonic acid, and it appears that all the allied facts of the circulation accord with this apprehension. In the complete range of physiological knowledge, there is apparently nothing so definitely and unmistakably understood as this, that while the red color of the blood is due to oxygen, the dark color is due to a like action of carbonic acid on the corpuscle. According to a long existing apprehension, this is an indisputable fact in the circulation. Instant derision * from all his professional fellows would befall him who should, even incidentally, so far forget the supposed grounds of certainty, as to think of the possibility of there being any element of misapprehension in the case. Accordingly, no work on Physiology fails, in descriptions of the blood, to represent most explicitly that it undergoes "two changes of color," the one of which is caused by the oxygen, the other by the carbonic acid. The one is supposed to consist in the conversion of venous blood, which is blood, the globules of

* This is why six years ago we feared to write on this subject, but we have now a far more complete knowledge of it than then.

which have a purple-red hue, into arterial blood, which is blood the globules of which have a bright red hue, and the second, to consist in the conversion of arterial blood into venous blood.

But in reality nothing, no phenomenon, of the character of the events we invariably name changes in the color of the blood, really takes place there; and though, perhaps, nothing can be more difficult, if possible, to obtain for such a rectification of a great error, any but a slighting and grudging acknowledgment, yet in my estimation there has come to light, in modification of the vast body of facts of science, embodied in its literature, no fact of equal importance with this, namely, that instead of two changes of color of the blood, there is but one color and one change in that color.

Seeing then that there are no facts in the course of the circulation, corresponding to this supposition that there are two changes of color of the blood, how shall the reader come to clearly discern the hitherto unperceived character of the facts?

Two "changes" affirmed, predicates two colors of the blood, and the supposition of those two changes is of a character which effectually precludes the apprehension that there is a color of the blood existing apart from these changes, since in themselves these changes are supposed to be precisely the colors of the blood, consecutively *produced* or effected by two agents causing first one and then the other of the two colors. For, these colors cannot be supposed to be two colors of the blood in itself, but only colors produced in the acts of change. These changes, we cannot suppose to take place in a *given* color, more or less lasting color of the globule; but only by and in the changes themselves. Thus excluding the apprehension of a given color, which if also admitted in our supposition would constitute three colors; namely, the two produced by the changes, and also a given color of the blood, not caused by the changes. In our present misapprehension we thus exclude the notion of a *change in a*

*color*, not *produced* as the consequence of the act of change, and affirm instead, two colors so *produced*, which supersede each other from moment to moment. This is what we name two changes of color. The defect and error of our misapprehension therefore consist in our having thought of two colors, purple-red and bright red as respectively the *products* of oxygen and carbonic acid, without the slightest apprehension (such as is involved in the truth of the facts,) of any color which itself was identified with the globule throughout any change. So that in an idea of these two colors there is an entire void of apprehension of any color not so produced.

But as this notion of two changes of color in the blood, supposes two distinct colors, in which the changes may occur, so it leaves totally unrecognized, that there is any state of this fluid in which there is a color, not the embodiment of a change and the immediate succession of a prior similar change.

It is manifest then, from these statements of our long standing notions, we have had no supposition that there is no color of the blood which belongs to the globule in itself, but only two colors produced ultimately in it; one by the oxygen, immediately followed by another caused by the carbonic acid; that is, that the globule is actually turned to a red color and afterward to a purple-red color, these colors being entirely due or given to it by the oxygen and carbonic acid. They are not, therefore, either of them "changed in," and *of* the other color, but two colors. There is neither room nor ground here, for the supposition that there is any color of the globule, nor any state of it, which is not comprised in one or the other of these changes.

But it is abundantly manifest here on a moment's reflection, that in the use of the word changes of color above, the character of a *change of color* is not truly represented at all. For a change of or in a color truly means, the subversion or "conversion" of the qualities of things, in some of which latter the change *consists*, and demanding subsequent conversion; but are simply a modification of speaking thus. In the single change which does occur, the blood globule does not *lose* any color in becoming red, nor has it any more color than it has in its purple-red hue.

But in our present apprehension of two changes, no change truly speaking is represented at all. For the latter means, and is the color or colored substance during its changes, an unbroken continuation of its existence throughout the apparent interval of its continuance, and this colored substance, will be found in such a case, neither to have lost nor suffered an *increase* or conversion of it, in the time of the change. This true change, specifies *one* color, in and by means of, or with which the change occurs. And the periods are change and unchange. But no such truth as this is represented in the current apprehension of "two changes of color." When we say two changes of color, therefore, there is an entire want or hiatus of the recognition of a truth, or what we suppose is not said, a fact of change. It is therefore certain that no fact which exists, is represented by the notion of two changes of color in the blood; and accordingly, what is the fact *is* represented by another, and the only true meaning of the term change of color.

What therefore we designate two changes of color is in reality only one color, namely, the purple-red, and one change in that. Not one change and another or second change, making two changes; and when we say the latter we undoubtedly mean by it two colors, implying that neither *one* of them is undergoing a change, but that the phenomenon is plural, and consists of these *two colors.* Here in reality a true change does not accrue to either color. Each color has the same status, and either is produced by different causes, namely, carbonic acid on the one hand, producing one, and oxygen on the other producing the second. The colors are plural, as the number of supposed producing substances are. But in calling these changes we apply the term without the slightest apprehension of its *true* import, and instead thereof mean by it something entirely different from this true meaning.

But let the reader for himself evolve some distinct conception of the supposition of two changes in color of blood. We are sure he will consult his own advance in rectification of error. This we know with entire certainty, that instead of this distinct conception of thought corresponding to some phenomenal reality, he will become aware of a mental hiatus, under this name of two changes of color; for this is all simply what the notion of two changes of color is. In other words, no such *two* changes of color are, while the *verbal* form affirmative that they are, is necessarily untrue. Cannot we then see the true meaning of a change and therein see the fact. Seeing this, we recognize the falsity of the meaning just before alluded to, namely, that of two changes of color, corresponding to which there is really no fact in the circulation.

But let us suppose the reader for the first time raising the conception of a true change. This conception, which is not involved in our present idea of two changes. Let us inquire with him what is a change? Does not an accurate definition of it imply some form of substance, which is not the change in itself, but the thing in which the change occurs. The change is what supervenes upon the face or in the quantitative constitution of the thing. It is no *new* thing added to the precedent sum of things; it is, in the case before us no *new* color created or produced to supply the place of the precedent color. It is in fact during the change no other than this precedent thing in whose color the change occurs. The change is not a *new* color produced or created. This above all things is palpably true, that in the case before us, a *new* color can neither be made or caused, because color exists only in the constitution of a substance or thing. But if you have your *colored* substance or thing, a change therein is readily understood.

Any supposition therefore of two changes, involves therein two color-producing agents, carbonic acid and oxygen. But in a true apprehension, namely, that of one change of color, there is no difficulty in understanding the fact that it is begun by the access of oxygen, and ends with the loss or release to the plasma.

Our present defect is that we do not recognize that the "return" to dark is not caused by carbonic acid, but is synonymous with loss of oxygen. This is the very source of our embarrassment on the subject. Instead of supposing that the carbonic acid changes the red to dark, we must recognize the latter as the proper organic hue of the globular substance, and the red as the change in it.

Any supposition of two changes necessarily places the purple-red upon the same basis as scarlet, it being *produced* or induced for the time being, precisely as the scarlet is by some current cause, namely, carbonic acid. Our notion now is that carbonic acid and oxygen, are substances each of which produce certain effects on the globule, the effect of one overcoming and superseding that of the other, and so on in alternation. Thus neither of them is a change on and in the other as a color, but they are simply alternate colors, produced by these two substances.

We have always supposed the blood to have two distinct colors, one in one part of the system, and a second in another part of the system. That it was dependent upon these changes for its colors, they being processes which made these colors, which otherwise would not exist. And that there was no blood during the round of the circuits, when one and the other of these changes, making its color there, was not in existence; so that the blood in the venous system was made to be dark red by a change, and that in the arterial system made to be bright red by another change.

Yet the slightest accurate attention to the facts, will inform us that this is an error. For the blood, or its red corpuscles must have a color existing from the first, a color which is that of the coloring substance. A color therefore not made by the carbonic acid it meets with, which is no part of coloring substance, nor by oxygen, which also is not its coloring substance. As this color, in the form of coloring substance in the constitution of the globule, exists in perfect union throughout with its other constitutive atoms, it not only must (and is) distinguishable wherever the globule appears, but whatever change in the globule occurs, must plainly be dependent on the existence of that color, must indeed be a mere change or *mutation* in that color; must be in short, a change in which that coloring substance furnishes all the color more or less, embodied in the transaction. Whatever change takes place, it will not cause the obliteration or destruction of this color, not only because this color must from first to last exist so long as the changes exist, in order to be the basis for the change; but because such destruction must necessarily consist of the complete disintegration of the constitutive substance of the globule. For in no other way could we get rid of this constitutive color or coloring substance, for the advent of a second one. But our present supposition of another color taking place in the globule, as one of the *two*, we suppose to form its *two* colors must be false, for in this event, of vacating one color of the globule to make way for another, the globule with its color, is destroyed.

Nothing is more plain, indeed than that this constitutive coloring substance of the globule must continue to exist intact with the globule, and it is not until the latter is destroyed or completely disintegrated that it can be dispossessed of its color. Moreover, this color or coloring substance, and it alone, is what makes the globule susceptible to the change of color we see, though we do not recognize its true character. This color is not a change in *another* color, as we erroneously suppose, but is the color in which the change takes place. Hence, *another* color, making *two* colors, than this constitutive color which is its colored substance, does not exist. And what we name *another* color is simply a change, a mutation in it. This mutation is neither a destruction nor conversion of the substance of this color substance, since it must continue undestroyed in order that the change in it takes place, not for once, but for any number of times. Were *this* supposed conversion or destruction once to take place, no possibility exists of its immediate restitution, re-making or re-creation.

It is evident from this, that there is a color of the globule which exists from the first to the last of its existence. Not a color unsusceptible of change, but one which is identified with the globules at any time wherever they are found. And that whatever change takes place, it cannot take place without this color, which must be the very substance in which it occurs.

Let us, however, assure ourselves of this latter fact, by considering the department in the blood of those globules without color, the white globules. It is manifest that whatever substance come in contact with these in the blood-fluid, they can not undergo any change of color. And why, simply because they are not colored. In short, they have no coloring substances *in* which a change can take place. A coloring substance, therefore, in the globule, its own substance giving it color is the one only condition of a change of color. More than this, the *sameness* of the color if the change is to take place frequently, must persist. It must be one color throughout, and the change will not constitute it of another or second color; much less, as we erroneously suppose, can this color be put alternately out of existence and in again, by any change.

This change takes place with the adhesion to the globule of the oxygen, and ceases with the loss of the oxygen to the fluid of the blood. During this event and only during it, is there any change of color, and only that portion of the blood which is in the arterial system has undergone it. This blood is bright red. From the moment it loses its oxygen in the capillaries until it again takes in a supply it is unchanged, and is therefore of a darker hue. This darker hue is its *un*changed condition. It is the constitutional color of the globule, and furnishes the very color which constitutes a change. Furnishes indeed the very color which during the change is bright red. It is itself no change in a color, nor is it the product of carbonic acid, for no carbonic acid enters the red-globule at any time.

It is evident that we have an ill defined notion-like state of mind, in the absence of any understanding of the truth of the case, by which we are *insensibly* duped, that during the changed state of the red-globules of the blood, there was something *not* there, which did exist before the change ensued. But in reality if there was something not there, it was something lost in the act of the change.

But if there really was anything of the nature of loss, if in that act there was any driving forth, decrement, destruction or diminution of what constituted the color in which the bright red ensued; in which the change consisted, how happens it that with *cessation* of this change, every destructive element of the globule. even to the least atom of its color, is precisely as it was before when dark, or in a state when not changed?

Let us dwell a moment upon the error of supposing this duplexity of *two* colors in the blood, for we can hardly fail to experience therefrom the rectification of the error; reason operates, on the least real apprehension of the case.

If we suppose that, when it is found to be red, the blood globules leave the color and parts from it, while coming in contact with oxygen, which it then is, namely, the venous hue, and the color which further on its road, it is again, what befalls this dispersed color? The oxygen carried by the globule cannot destroy it, because it is carried to the capillaries. If we suppose the oxygen as soon as received to destroy the color, it must in doing so, go with it, thereby leaving the globule minus its color; for this destruction it can only do by combining with the color substance and taking it to itself, thus leaving the globule to go on its way without either. Not only this, but in the supposed act it must have disintegrated the globule itself, by disuniting or decomposing the union of its compound particles, and abstracting therefore those which constitute the coloring matter. For in no other way can we imagine (of course erroneously imagine) this leaving of the color of the globule, to occur. If on the other hand we suppose, that the color it is when entering the lungs, is not left by the globule nor destroyed, the same color must be that of the globule in leaving the lungs, when it is arterial red; and even though it has just received oxygen, if the color was not thereby either left behind or destroyed, the red of the arterial hue cannot be *another* color, because the event it has undergone, namely, the in-taking of the oxygen, cannot introduce another color, in virtue of its own particles, without disposing of that the globule is before entering the lungs, and this it has not done. Nor can it make a new or other color by instant "conversion" of any portion of the substance of the globule, for in order to dispossess it of its venous color it must exert a destroying action on the globule in order to eliminate the color substance, an action in which it must become united to that substance, and thus united leave the globule with it.

Nor can the oxygen, the globule remaining undestroyed, convert in any sense its substance into *another* color. What it can do in that case—the globule having the color substance with which it entered the lungs before leaving them—is limited to some *present* influence upon that very coloring substance, remaining intact and undisturbed as an indivisible part of the globule substance and of its make up.

What is the character of this influence, viz.: the influence the oxygen exerts, marked by the bright red as contrasted with the dark red? We have seen, that it cannot consist in the introduction nor formation of another color in the globule by any mode of conversion or composition, of substance therein, since the coloring matter composing it, these cannot be either destroyed by any action of any kind, which does this while it forms a part of the globule, nor be driven forth from the globule. Since in the latter case the globule itself would be destroyed, its component atoms altogether going into a state of dissolution. And if another color cannot be made in, and substituted for the previous color substance, neither by destruction of it, nor by conversion of substance by oxygen, there is no other way we can imagine (even erroneously,) *another* color to take the place of the previous color. If it does not occur, another color to the globule is neither produced nor made in it, and our supposition, that such an event takes place, is entirely erroneous, and the color substance; and hence color, which was in the globule when venous, is the same as when arterial.

But to answer our question, of the nature of the influence which the bright red color denotes. The very statement of the foregoing makes it evident,—1st, That this can only be very limited, and—2d, That it must be limited to and consist wholly in some present change in the coloring substance in question, which the globule has before receiving oxygen, and not in any new production of or *another* color, which can only be possible by production of a new or other color substance. This is perfectly plain, as the first is not destroyed, nor gives way to any other. A change, however, does take place, since the blood *is* bright red and *was* dark red. Yes, precisely, this word change, truly signifies the exact character of the influence of the oxygen. But then this change, denoting what the character of this influence is does not consist in any new *production* or integration of color: of another color. In our present dearth of distinct conceptions on the subject, we use it most erroneously; use it as descriptive of the formation of a second color, and hence, as (erroneously) indicative of a change which consists in this new *production* of color, while in reality it truly represents the fact, and is properly used only when it indicates some mere passing or present mutation in color substance, already there.

*This* change then is really what takes place. And what takes place, is therefore a phenomenon, not ensuing upon either the *destruction* or driving out of the color substance or color of venous blood, but of its undisturbed (at least by this event,) continuance in the formation of the globule. The very existence and character of this change is wholly dependent upon this continuance of colored substance as before; upon the maintenance of its identity throughout the entire existence of the blood globule. So that without this persistence of its identity, without destruction, and without substitution, of *other* colored substance, without renewal from the moment the globule is formed until it dies, this phenomenon of change of color, could not occur in the movement of the blood.

One invariable (erroneous) supposition of more than a change of color in the blood, namely, of two changes of color, is also a pure fiction. This in the nature of the case not only is not, but *cannot* be, and the error is so entirely obvious that we are surprised that any person who has given the least intellectual attention to the case, could continue to affirm it. Yet it is our invariable affirmation. What are the facts involved in the occasion of this egregious fallacy?

Suppose we remove one of these changes of color. Suppose for instance the one change the blood does undergo from the access of oxygen to its globules, does not take place. Does a change remain? Does the other one of the two changes take place? Suppose the blood is of the venous hue, one of the colors of the two changes, and does not become red, or change? Would another change exist? Obviously not, it would continue to move *without* any change. No change whatever remains. In omitting the one change by oxygen, we have omitted all the changing of color there is in the case, for there is but *one* change. How is it then, that we invariably do, and have from the first, without a single dissentient voice or doubt, continued to affirm, and what is more *believe* in, *two* changes of color of the blood, without ever dreaming of the perpetual fallacy it denotes?

The fallacy is easy to expose, and our failure all this while to see the truth, demonstrates in a way no demonstration can excel; how utterly vacant the minds of men may be for ages on a given subject, the whole while they are accrediting themselves, a perfect knowledge of it. It shows, moreover, with equally overpowering force, what utter fallacy and falsity is involved in any apprehension of a subject, which is confined to mere observation or to what is seen, irrespective of the exercise of the reason. We said the fallacy in question was easy to expose.

We have seen that if the blood made its round, without the arterial hue ensuing from time to time,—which is but *one* change—there would be no change. It would be throughout the circuit and for any possible number of them *un*-changed, or continuously of the venous hue. In other words, let it undergo no change in color from oxygen, and no change occurs in it. It is continually venous, which is its color without any change; its color unchanged by oxygen. Being unchanged, when the oxygen does not change it, how then is it we call this unchanged state, one of *two* changes? When it occurs not continuously, but *between* the repeated changes due to access of oxygen?

But we may still say, this present state of the blood is another change. How is this?

Let us then alter the previous supposition. Let us not omit the change as we supposed by removing the oxygen, or preventing its entrance into the blood at the lungs,—the omission of the change consisting entirely in this—in anticipation of the change, or *before* it takes place, but omit it or do precisely the same thing with it during the existence of the change, while the blood is away from the lungs; that is, let us withhold the oxygen from the globules.

Shall we not then inevitably find the blood in precisely the same condition of color,—dark red—and without change, that having been discontinued, though just done, by removal of its cause, oxygen; that we found it to be, when we omitted the change by preventing its cause—oxygen from making the change in the lungs? Indubitably!

What is this answer then but a statement of the fact, that if we omit one change, the one which occurs, by removing the oxygen which causes it from the lungs, or removing it from the globules after they have passed from the lungs, whereby we omit the change, whereby we do not permit it to take place during one of the times of change, and whereby we do not permit it to continue, or any longer to take place for the time being, that the blood is without change, and its color an unchanged color, the dark red?

How then can we call this state of the blood, its dark red state, another change? No greater error can exist than to suppose this dark red hue is a change.

But we may again say, this dark color is due to carbonic acid, hence it is change. It is sufficient to say, that no such thing takes place. No carbonic acid leaves the blood-fluid to add itself to the globule. We now affirm that from being, not in the globule itself, but in the liquor sanguinis in which the globules are suspended, the carbonic acid from the former enters into the latter. And the color of the globule, and hence, of the crude blood, we suppose to be due to this carbonic acid. The blood is darker than it had been a few seconds before; it was then scarlet. We suppose it has undergone a darkening. True, the blood is darker than it was, but this only because what it was before in hue was a changed state of, or mutation of, this same dark color, which was in reality what was before the change, and is now the same hue again, before the next change by oxygen in the lungs.

At a certain period of the progress of the blood, the red corpuscles present a darker color, seen as that of the whole mass of the blood, than the same corpuscles were of just before they reached the capillaries. This we *imagine* is due to their receiving carbonic acid from the liquor sanguinis. According to this imagining they undergo some "conversion."

We further imagine this conversion to be reversed soon afterward, *i. e.* in the lungs; for here, the converting substance leaves the globular substance. In this imagining there is nothing, which in the least hints at what the supposed conversion *consists* in. According to the supposition, it merely lasts for a few seconds, after which the blood, according to what is *seen*, is as it was before conversion, or *unconverted.* Seeing the void of any notion of what it consists in, we inquire what is the character of this imagined conversion? Leaving the further citation of our embarrassing errors and defects, in order to comprehend the truth in answer to this question, we must respond that any conversion of the globule must be either of its form, substance or other attribute, if it have any. Can we say that either of these have taken place? Certainly not; because any real conversion, must be in effect more or less lasting. It must consist of a greater or less difference in kind or quality of substance, from what it was when the act of conversion set in. It must, in short, exist on some character of embodiment, form or substance, which supersedes or comes into existence instead of the character which existed before, which are thus put out of existence. But nothing of this nature, which alone constitutes a conversion; and hence, no true conversion takes place in the case of the globule, as we mistakenly imagine it does, during the interval the globule passes between the scarlet and dark color.

But not only does no conversion as we imagine to take place, no change of any character takes place. For the globule immediately after this supposed conversion is again light. This according to our false supposition is after it has taken into its form carbonic acid, and occurs upon the loss of the latter. But in any cause of change, if due to carbonic acid, when the latter *returned* to its previous relation of separation from the globule, the latter must *thereby* be also returned to its previous hue, to the hue the carbonic acid is supposed to have changed it from. But our supposition does not involve the admission of any such fact. It includes no such affirmative, which it is constrained to do, by the nature of the truth, if the truth lie in our supposition. On the contrary, we affirm that the hue of the blood, immediately after the loss of carbonic acid, is not *a return* to that we suppose the carbonic acid has changed it from; but is distinctly a change made by the accession of oxygen. And this affirmation embodies precisely all there is of our present supposition. Having supposed that carbonic acid passes from the blood by air, to which it was exclusively confined before, and the globule; and that on its entrance the globule becomes of that color, which gives both together the dark hue of blood, we make not the slightest, even imaginary, or any exception of any further phenomena on the subsequent separation of the two. We affirm the separation at the lungs, and we are constrained to acknowledge that the supposed change only lasts till the lungs are reached. This admission embodies the unexpressed acknowledgment that the dark only commenced *by* the presence of the carbonic acid in the globule, and lasts during *that presence there.* But if the dark hue is made (as we say) by that presence, and continues only during that presence, then it stands to reason, if it fails of a longer continuance of that color, this must and can only be consequent on a withdrawal of that presence. So that if carbonic acid does enter and does leave the globule, meanwhile causing it to be dark by its presence, it must necessarily *by* leaving of the globule, by withdrawal of its presence from it, withdraw the cause of its being dark; that cause being itself. The logic is inexorable; it is the only logic there is in the reason of the case, and the only reasoning which embodies the truth, if it be as we suppose.

Yet literally all we have represented in the form of our supposition on this subject, contains no word of reference, either imaginary or any other, as to the carbonic acid, its loss or its gain, as having anything to do with the state of the globules when they *become light.* Yet the color which we supposed to be caused *by* the carbonic acid in the globule, *must* when the carbonic acid goes out of it, no longer continue. In short, if it changed the color, and changed it by *being in* it, the change can have no existence any longer than it is in it. The moment the carbonic acid gets out of the globule, the color it caused by being in it, must go out of existence with the retiring of its causes. The dark color, in short, if the case be as we suppose, must go out or cease with the going out of what induces it while in the globule.

But we hitherto entertained no supposition, that such an occurrence as the going off of this dark color takes place, and have used no word in our representations, which is indicative of this notion. Yet such must be the case, if it had as we suppose, its advent with the supposed coming into the globule of the carbonic acid.

But if no dark color is in the globule, nor is voided by the going out of the carbonic acid, and no change to light is effected by its going out (since that change is due to oxygen), then it must be left or continue that of the globule, until the oxygen enters it. Taken by itself, this is of course what we are ready to acknowledge, in accordance with our present supposition, whatever the truth may constrain us to when operating a rectification of that supposition, by means of showing what else it does not contain or is deficient in the assertion of, and which should be true, if *it* be true. Accordingly, we admit that the dark color of the globule, and hence, of the entire blood does not go off, because carbonic does, but exists when the oxygen enters the globule. This latter act and substance it is which induces the light color.

But if the oxygen find this color of the globule existing, what can the scarlet color be but a change in that dark color. The former ensues upon the access of the oxygen. The scarlet color is a change of this prior hue. It is the color the oxygen finds the globule to be, *upon* which the change supervenes, and which it leaves the globule as being, when this change terminates. What other color can possibly be imagined to exist here, which is the second of two colors? Since the scarlet is but a *mutation* of the darker hue.

Let us fully apprehend this point. We say then, the supposed darkening of the blood by carbonic acid, does not occur. If now, instead of supposing what contrary to the fact we hitherto have, that there are two changes of color, two colors of the blood, let us again suppose that the blood "experiences no change of color" (Bernard's words). In this entire absence of change, would there be an absence of color? Certainly not, since the absence of that would preclude all possibility of change. There is therefore no absence of color. Though this white globule color is supposed here to be unchanged, or to experience no change of color, it must of course be that (*color*) in and of which the change occurs. What must we suppose now, in order to present a change in this color. The very synonym of change is some passing affection or modification in the character of a thing, and in the present case this passing change is owing to the oxygen of the air. If this affection was not passing or transient had no immediate limit or cessation we should have a persistent color, which persisted throughout, succeeding the first, though not *additional* thereto, nor another one of two coexistent colors, because that is impossible. But instead of this, this change in the color abides as first we said, "experienced no change," lasts only so long as the oxygen is carried by the corpuscle. It lasts therefore during a certain interval in the circuit, namely, in the arterial system, and during the rest over it lasts no longer, we have the blood experiencing no change. Surely we have neither two changes nor two colors here, but one color, having intervals of experiencing a change, and between for the rest of the time, when it experiences no change. Now the color in this latter period, is precisely what it would be continuously experiencing no change, if the change did not occur. It is the venous hue, the change is the arterial hue. So that this at first persistent color, experiencing no change has a passing change in it.

What divides the blood into periods of color is the change or brightening, and that alone. The arterial tissue is precisely like a syphon which divides the otherwise wholly undivided tissue in two. One occupied by the purple-red, the color of the blood when "experiencing no change," and one by the blood experiencing a change of this same color. But the cessation of the latter is we see, not at that it is superseded by another induced by carbonic acid (for there is no other color in the case), but is because of the taking place of the period over which blood is of the color when experiencing no change. What therefore we suppose to be one color, of what we call two colors or changes, is simply but one and the same color in a state of change, and experiencing no change. In the one bright-red, in the other purple-red.

This change and no *other takes place*, for when this change is over it is the very negative or *privation* of the event which ensued by entrance of oxygen, and hence what exists is the continued color of the globule itself.

Let the reader ask himself if when the oxygen has joined the globule, there is any more or less globular substance or coloring matter; or any more or less color. If he can possibly detect how much *more* color there is in the red than in the purple, or how much *less?* But he must all the while remember that this more or less color defines the character of the change pertaining to one color, and is only a difference by comparison between *two* colors; between one and another color. Let the reader think of *where* this purple-red color is, while the bright red exists; it is not evacuated or released. If it was, it must be replaced by what we have called the other color. What substance can do this? Not the oxygen alone, but only some soft substances equivalent in density, mass, character and color to that displaced. In short, it must be a substratum of the same characters as those of the globule, if not its form, to replace that erroneously imagined to be removed.

Moreover, if carbonic acid did enter the globule, it could make no such change as we erroneously suppose.

For, we have seen that while the blood is of this dark red color, the oxygen comes in contact with the globules and changes it, and that the color or colored substance is precisely what the change occurs in. And furthermore, we have seen that if we omit or remove the oxygen, the blood is precisely of the color it was, *i. e.* dark red. With the removal of the oxygen there is a cessation of the change, the color thereupon being precisely what it was and invariably what it is when the change does not exist. What participation has carbonic acid or any other substance here?

How then can we attribute this color to carbonic acid and call it a change of color due to it, when that substance has no participation in the case, and when the dark color always exists in the very anatomy of the globule, when not changed by oxygen.

In these representations we have gone over the ground briefly. But the whole facts may be thus summed up.

1. Change occurs in the color of the blood, already existing and not arising from *any* present or immediate cause in the blood itself, but embodied in the formation of the globule itself, and lasting from its formation to its death.

2. This change occurs in the always colored substance of the globule, which is dark red, and is caused by the contact of the oxygen of the air with it.

3. This change occurs an indefinite number of times, having a certain duration, which is while the blood is in the arterial system.

4. Between these durations or times of change, there is corresponding times when the change does not exist, the oxygen carried by the globule having been distributed throughout the plasma.

5. No change but one many times repeated exists in the blood; no *two* colors exist in, due on the one hand to oxygen, and on the other carbonic acid; but only one change of a color exists, due to oxygen.

---

The extraordinary importance to physiology and pathology of correcting this fallacy of two colors and two changes of the blood, which has never been questioned from the beginning, will be perceived when we say, that it enters into all our theories and conceptions of the behavior of the blood, and to its full extent constitutes them all alike *erroneous.*

As a direct prompting of these fallacies we suppose the blood in the veins to be a fluid of widely different character and properties from that in the interior. Notwithstanding the fact that the latter was but an instant before venous, and has undergone no change except the addition of oxygen, wholly confined to the globules until it has advanced to the general capillaries.

Of the exact character of the change which takes place on accession of oxygen, we shall have something to say when time and opportunity occurs.

In order to save the reader the necessity of further study of the subject in hand, to appreciate those contradictions, we state that it is now known that the purple-red (or venous hue) of blood is no changed color *from* any other color—it being itself the very color in which the change occurs, which is the bright red (arterial hue), on access of oxygen. And this we may also say, stands to reason. The blood does not therefore *change* from "red" to "black," as is contrary to the truth represented, for since the red is itself a change *of* and in the darker hue, this last cannot be a *change in a change.* The truth being, that this darker hue is the constitutional color of the globule, in which intervals of change to bright red occurs. This change is supposed to be due to carbonic acid. One, contrary to knowledge *learning*, represents that certain experiments of Priestly showed that—"Venous blood is not changed in color by nitrogen, hydrogen or carbonic acid, while all these gases, *by displacing oxygen*, will change the arterial blood from red to black."

But these experiments did not show, what contrary to the fact is supposed. What they did show was, that oxygen left the globule. It was supposed to be displaced, because it was not imagined that oxygen will itself leave the globule, apart from any "displacing" process. When we say, therefore, that Priestly's experiments did not prove what is represented, the truth meant is, not that oxygen will not leave the globule, but that this event was not one of "displacement," by any of the other gases named.

So too, in saying that neither of these gases changed the blood from red to black, we are simply saying that this purple-red or "black," was not a change in any preceding color, because that preceding hue was only this same purple-red in its change or brightened interval,—but was simply a cessation of its changed state by loss of oxygen.

The facts, therefore, in these experiments are, loss of oxygen not by any other gas entering the globule and displacing it, and cessation of change of hue during arterialization.

We must now further inform the reader that experiment discloses the fact that the coloring matter of the globule will regularly take up and surrender or release the oxygen of the air, many times in succession, where neither of the gases named have access to it, or are permitted to be present.

By these experiments it is ascertained that the relation of the oxygen taken from the air, to the globule is nearer that of the elements to a compound, but one in which it takes up and carries oxygen forward into the system; hence, the desiccation there of the oxygen from the globule, is not in any sense compulsory, nor demands the intervention of any other substance, but is simply one followed by the oxygen entering into combination with the materials of the plasma, through which it has been passing, and to which it is the business of the globule to distribute it.

Finally, if by this we are not fully satisfied of the truth in the case, that there are no two changes of color in the blood, and persist in regarding the dark red as the consequence of a change, let us suppose we omit this (erroneously) supposed change, leaving the blood red. What change remains; no change can take place. The blood is continuously red. We have as before omitted one of the supposed two changes, and no change remains, as before shown.

So that in either case, if we omit one of these supposed to be *two* changes, we have no change instead of the one which must remain, if there were two in fact.

This supposition of two changes, therefore, must be erroneous. For the dark hue, that which we erroneously suppose to be the present effect or consequence of another change, is only a *recurrence* between each time of repetition of the one actual change of the constitutional color of the globule. For as the former phenomenon, namely, the bright red, is one entirely based upon this dark color which inheres in the globule, so at all times it must exist in order that the phenomenon of change may occur.

Color displaced or otherwise disposed of, the reddening which is a phenomenon in it, could not occur. The whole of the case, which we by one misapprehension make a mystery, lies in the fact, that as this phenomenon of change, this reddening, takes place repeatedly, time after time without number, each time being a brief period, and each time followed by an interval which continues till the next period of change commences, the dark red is only seen in its unchanged state between these times of change. And the color during these intervals of its unchanged state. we suppose to be produced by a post-reddening effect, caused by *carbonic acid.* The error is not alone, that no carbonic acid enters the blood globule, but *if* it did, it could not make the color, which must inhere in the globule, in order that the phenomenon of reddening can take place at all.

